# Dietary Supplementation of Dried Grape Pomace Increases the Amount of Linoleic Acid in Beef, Reduces the Lipid Oxidation and Modifies the Volatile Profile

**DOI:** 10.3390/ani9080578

**Published:** 2019-08-19

**Authors:** Andrea Ianni, Alessio Di Luca, Camillo Martino, Francesca Bennato, Elettra Marone, Lisa Grotta, Angelo Cichelli, Giuseppe Martino

**Affiliations:** 1 Faculty of BioScience and Technology for Food, Agriculture and Environment, University of Teramo, via R. Balzarini 1, 64100 Teramo, Italy; 2 Department of Medical, Oral and Biotechnological Sciences, “G. d’Annunzio” University Chieti-Pescara, Via dei Vestini 31, 66100 Chieti, Italy; 3 Istituto Zooprofilattico Sperimentale dell’Abruzzo e del Molise “G. Caporale” Via Campo Boario, 64100 Teramo, Italy

**Keywords:** agricultural by-product, bovine meat, polyunsaturated fatty acid, lipid oxidation, volatile compound, biogenic amine

## Abstract

**Simple Summary:**

The grape pomace is the main solid by-product of the oenological industry, and represents a rich source of potent bioactive compounds. In this study we demonstrated that its inclusion in bovine diet resulted in a significant increase of linoleic acid concentration in meat, a condition which led to a positive increase in the polyunsaturated fatty acid:saturated fatty acids (PUFA:SFA) ratio. Despite the greater predisposition of PUFA to oxidation, an interesting improvement in the oxidative stability of meat was evidenced, presumably as an effect of the antioxidant activity performed by the bioactive compounds of which the grape pomace is rich in. This finding was also confirmed by the analysis of volatile compounds which highlighted a reduction of hexanal in meat samples obtained from animals fed the dietary grape pomace supplementation. Overall, the present study showed a viable way for the recovery and the valorization of the main by-product of the oenological industry.

**Abstract:**

The aim of this study was to evaluate the effect of dietary supplementation with dried grape pomace on beef quality. Ten Friesian calves were divided into two groups, a control group that received a standard diet, and an experimental group that was administered the dietary supplementation. At the end of the 75 days of the trial, animals were slaughtered, and meat samples analyzed for physical and chemical properties, fatty acids composition, lipid oxidation, volatile compounds, and biogenic amines. The fatty acid profile resulted affected by dietary supplementation, since an increase in concentration of linoleic acid was observed. Furthermore, a reduction of lipid oxidation was found in the same samples. With reference to volatile compounds a reduction of hexanal and an increase of 2-3 octanedione was evidenced, while no effects were induced by diets on the synthesis of biogenic amines. The grape pomace exploitation as a dietary supplement in bovine diet did not have negative effects on the quality of beef and showed the potential to extend shelf life due to marked improvement in oxidative stability. Overall, the present study showed a viable way for the recovery and the valorization of the main by-product of the oenological industry.

## 1. Introduction

The management of agro-industrial by-products represents an issue of great importance for its environmental and economic impact. Based on the reports disclosed by the International Organization of Vine and Wine (OIV), in 2016 the world grape production was over 75 million tons. The wine industry, which uses half of the entire grape production, is responsible for the production of a considerable volume of solid and hardly degradable residues (approximately 20% of the total processed grapes) which are normally used as fertilizer or simply discarded. Grape pomace (GP) is the main solid by-product of the oenological industry, and represents a rich source of potent bioactive compounds, especially polyphenols (2%–6.5%), such as phenolic acids, simple flavonoids, tannins, and proanthocyanidins, to which a wide range of biochemical activity is attributed [1]. Flavonoids have the capacity to act as powerful antioxidants by scavenging free radicals and reducing oxidative reactions [2]. Furthermore, the biotechnological potential and the remarkable nutritional and nutraceutical properties of many of these compounds has been associated to beneficial effects in humans, especially in the treatment and prevention of several chronic diseases, including cancer and inflammatory events [3]. For these reasons, and in consideration of its low cost and high fiber concentration, GP received in the last decade increasing attention as an alternative feed ingredient to partially replace fodders for all the livestock and especially for ruminants. 

The dietary integration of this by-product in lactating dairy cows showed to induce many benefits on chemical and nutraceutical properties of cow milk and cheeses; furthermore, interesting variations in the aromatic profile of ripened cheeses were evidenced [4,5]. Szczechowiak et al. (2016) investigated the effects of dietary supplementation of matrices rich in condensed tannin on the fatty acid (FA) composition in samples of rumen and milk obtained from dairy cows, finding an effective modulation of rumen biohydrogenation (BH) and fermentation. In particular, the inhibition of the last steps of BH with the consequent increase in concentration of vaccenic acid (C18:1 *trans*-11) was observed [6]. Buccioni et al. (2015) reported a study in which the specific inclusion of chestnut tannin in the diet of grazing ewes induced an increase of milk production and proved able to improve the effects of dietary linseed supplementation, resulting in milk with a greater concentration of α-linolenic acid. [7]. Nudda et al. (2015) evaluated in sheep the effect of dietary inclusion of grape seed flour, in combination or not with linseed, on milk production, immune status, and hepatic and renal metabolism of animals [8]. Recently the analysis of the whole-transcriptome of Friesian calves fed with a GP supplemented diet was also performed, observing interesting variations in the pathway of the cholesterol biosynthesis, which appeared to be consistent with a reduction in both serum cholesterol and lipid oxidation in the carcasses [9]. With specific reference to meat, Gómez-Cortés et al. (2018) investigated the effect of GP in ewes’ diets studying the effects on quality and fatty acid profile of meat samples obtained from their suckling lambs. They found that GP integration did not induce any negative effect on the carcasses but improved the water holding capacity [10]. Aditya et al. (2018) studied the effect of GP supplementation on growth performance and meat quality of broilers. In this case, the dietary GP intake improved the meat quality without affecting growth performance, nutrient digestibility, and carcass traits [11]. 

In spite the growing interest in the use of GP as integrator in different animals, and the interesting results mostly concerning the chemical composition of milk and dairy products, there are a lack of studies investigating the effect of this by-product on bovine meat. Hence, this study was carried out to elucidate the effects of GP on growth performance, meat quality, lipid oxidation, volatile compounds, and trace compounds in beef.

## 2. Materials and Methods 

This study used animals and data from commercial farms which were handled following the national legislation on animal welfare (DL n. 126, 07/07/2011, EC Directive 2008/119/EC), and then slaughtered complying with the EU Regulation 1099/2009 on the protection of animals at the time of killing. For the purposes of the study, the animals have not undergone breeding practices other than those normally envisaged and for this reason no other ethical declaration was considered necessary.

### 2.1. Animals, Dietary Treatments, and Sample Collection

The study was conducted in a farm located in central Italy (Casoli, CH, Abruzzo), in which 10 Friesian male calves, selected on the basis of weight (100–120 kg) and age (11–12 weeks), were randomly divided into two groups of five animals each; therefore in this study five replicates (*n* = 5) were considered for each treatment. The control group (CG) received a standard diet while the experimental group (EG) was fed with the same diet but supplemented with 10% (on dry matter basis) of dried grape pomace (DGP). Animals had 60 days of acclimatization period, in which they received a basal diet mainly consisting of alfalfa hay, whose composition is reported Table 1, with the addition of a custom-formulated concentrate offered ad libitum. 

The acclimatization period was then followed by 75 days in which the CG kept receiving the standard diet, while the EG animals received the DGP integration. Ingredients and chemical composition of the CG-supplement and EG-supplement is reported in Table 2. Dry matter (DM; method 930.15), crude protein (CP; method 954.01), fat content (by ether extract (EE); method 920.39), crude fiber (CF; method 962.09), and ash (method 942.05) were determined according to AOAC International (1990) [12]. Neutral detergent fiber (NDF) and acid detergent fiber (ADF) were instead evaluated following the procedure proposed by Goering and Van Soest (1970) [13]. 

During the 75 days of the trial, the feed intake was monitored for each animal at the beginning of the experimental period and weekly until the end, for a total of 10 evaluations. The calculations were performed taking into account the refusals of diets offered to the male calves.

The GP derived from red grape (*Vitis vinifera* L.) was obtained through a process that involved a preliminary phase of fermentation and subsequent treatment with steam for the purpose of eliminating ethyl alcohol. Subsequently the by-product was treated with water at 90 °C in order to recover the tartaric acid, and only at this point was performed the drying followed by the production of flour. The DGP used for the feeding supplementation as well as diets were characterized in terms of total phenolic compounds (TPC) according to the Folin–Ciocalteu method [14]. The fatty acid profile of DGP and diets administered to CG and EG was also analyzed (Table 2) according to Castellani et al. (2017) [15]. 

At the end of the experimentation, all animals, ageing about 7 months, were slaughtered in a commercial abattoir. The carcass of each animal was weighed and, based on the evaluations of the live weight previously performed, the carcass yield was calculated. The front quarter of each carcass was preserved following the protocol normally adopted in the commercial sphere, i.e., leaving the meat at a controlled temperature of 4 °C, covered by a film to avoid contact between the carcass and the surrounding environment. Meat samples were collected from the *longissimus dorsi et lumborum* muscle between the 7th and 13th rib, and, in order to evaluate changes in the chemical composition and quality attributes due to the storage at 4 °C, the meat sampling took place after 3 and 7 days post mortem, paying attention every time to perform the sampling only after having eliminated at least 2 cm of the most exposed layer of meat. Except for pH, which was evaluated directly at the slaughterhouse, the analyses described below concerning color, drip loss, cooking loss, chemical composition, and fatty acid profile, were performed only on samples collected after 3 days from slaughter, while lipid oxidation, volatile profile, and biogenic amines were evaluated both after 3 and 7 days post mortem. Some of these analyses (color, drip loss, cooking loss, and lipid oxidation) were performed immediately after sampling, while the rest of the samples were packed under vacuum and frozen at −20 °C waiting to be subjected to the remaining investigations.

### 2.2. Evaluation of Meat pH and Color

The sample pH was measured using a portable pH meter equipped with an electrode (Crison, Barcelona, Spain) which was adjusted for muscle temperature before being inserted approximately 6 cm into muscles. Measurements were taken in triplicate from the loin after 45 min and 48 h from slaughtering. Before analysis the pH meter was calibrated using standard phosphate buffers (pH 4.01 and 7.00) and after each measurement the electrode was rinsed thoroughly with distilled water. 

Color measurement was performed by the CIELAB system. L* (lightness), a* (redness), and b* (yellowness) values were registered from a transverse section of the *longissimus dorsi et lumborum* using Minolta-CR 300 (Minolta Co, Osaka, Japan), with a D65 illuminant, 10° standard observer angle and 32 mm aperture size. The instrument was calibrated before each series of measurements using a white tile (L* = 100) and a black glass (L* = 0). Each sample was measured in three different areas at 3 days post slaughter.

### 2.3. Drip Loss, Cooking Loss, and Chemical Composition of Meat Samples

In order to determine the drip loss in meat, samples of about 2.5 cm of thickness and approximate weight of 80 g were inserted in an expanded bag and left at 4 °C for 48 h; after that the meat sample surface was lightly blotted with a paper tissue and reweighed. Drip loss was then expressed as a percentage of the original sample weight. 

The cooking loss was evaluated on meat collected at day 3 post mortem. Samples of 80 g and about 2.5 cm of thickness were cooked to a core temperature of 75 °C (Minitherm HI8751 temperature meter and probe, Hanna Instruments Ltd., Bedfordshire, UK) in a water bath (Grant Instruments Ltd., Barrington, UK), tempered at room temperature and left to cool at 4 °C overnight. Cooking loss was then expressed as a percentage of the original sample weight.

For the evaluation of chemical composition, meat samples were preventively minced by using a 5 mm plate meat-grinding machine. Then were performed analysis in order to obtain information on moisture, ash, and fat content [16]. 

### 2.4. Fatty Acid Composition and Lipid Oxidation

The Folch method was used for the extraction of total lipids [17]. About 5 g of minced meat were homogenized in Ultra-Turrax T-25 in 90 mL of Folch solution. Samples were stirred for 6 h at room temperature and filtered overnight in presence of sodium chloride; the chloroform phase was then evaporated to dryness with a Strike-Rotating Evaporator (Steroglass S.r.l., Perugia, Italy) at 38 °C. From the total fat obtained for each sample, 50 mg were weighed and mixed with 5 mL of hexane containing 100 µL of sodium methoxide and 100 µL of methanol, in order to obtain the fatty acid methyl esters (FAME). Detection of FAMEs was performed by a gas chromatograph (Focus GC; Thermo Scientific, Waltham, MA, USA) equipped with a capillary column (Restek Rt-2560 Column fused silica 100 m × 0.25 mm highly polar phase; Restek Corporation, Bellefonte, PA, USA) and a flame ionization detector (FID). Hydrogen was used as carrier gas. Peak areas were quantified using ChromeCard software, and the relative value of each individual FA was expressed as a percentage of the total FAME. The identification of individual FAME was performed by comparing the retention time of the standard mixture FIM-FAME7-Mix and individual C18:1 *trans* 11 (Matreya LLC, State College, PA, USA). The value of each FA was used to calculate the sum of saturated fatty acids (SFA), monounsaturated fatty acids (MUFA), and polyunsaturated fatty acid (PUFA).

Lipid oxidation was measured by the thiobarbituric acid reactive substances (TBARS) method following the procedure previously reported by Ianni et al. (2019) [18].

### 2.5. Analysis of Volatile Compounds

Minced meat (3.5 g) was mixed with 10 mL of saturated NaCl solution (360 g/L) and 10 µL of internal standard solution (3-methyl-2-heptanone; 10 mg/kg in ethanol) was added. VOC were extracted from the headspace with a divinylbenzene-carboxen-polydimethylsiloxane solid-phase microextraction fiber (length: 1 cm; film thickness: 50/30 µm; Sigma-Aldrich, Milan, Italy) with an exposition time of 60 min at 60 °C. Then, the extracted VOCs were thermally desorbed into the gas chromatograph (Clarus 580; Perkin Elmer, Waltham, MA, USA) coupled with a mass spectrometer (SQ8S; Perkin Elmer, MA, USA) and equipped with an Elite-5MS column (length × internal diameter: 30 × 0.25 mm; film thickness: 0.25 µm; Perkin Elmer). The thermal program and the recognition of the individual VOCs was performed as previously described [19].

### 2.6. HPLC Analysis of Biogenic Amines

The determination of biogenic amines was performed through previous acid extraction and derivatization with dansyl chloride, by following a slightly modified methodology previously reported by Schirone et al. (2013) [20]. The chromatographic system consisted of a HPLC (Varian, Palo Alto, CA, USA) connected to a variable wavelength UV-VIS detector and equipped with a Supelcosiltm LC-18 column (25 cm × 4.6 mm, 5 μm; Supelco). Biogenic amines were separated using a mobile phase constituted by ultrapure water (A) and acetonitrile (B). The initial mobile phase contained 50% A and this percentage was linearly decreased up to 10% in 19 min; after that, the content of A increased up to 50% in 3 min and this condition was maintained for 8 min until the end. The flow rate of the mobile phase was 1 mL/min and the column temperature was set at 40 ± 0.1 °C. The peaks were detected at 254 nm and the identification of the biogenic amines was based on their retention times. Tryptamine, 2-phenethylamine, putrescine, cadaverine, serotonin, and tyramine were used to obtain the calibration curves (peak area versus concentration), that were linear in the range of concentration between 0.5 and 50 mg/L. The calculated lines of regression were used to compute the amount of the analytes through interpolation, by using the method of the internal standard (1,7-diaminoheptane).

### 2.7. Statistical Analysis

All experiments were performed in triplicate and the results were reported as mean ± standard deviation. The SigmaPlot 12.0 software (Systat software Inc., San Jose, CA, USA) for Windows operating system was used to analyze the statistical significance of the differences between the averages for each group (ANOVA and Student’s *t*-test); *p* values lower than 0.05 were considered statistically significant.

## 3. Results

### 3.1. Physical and Chemical Properties of Beef

As shown in Table 2, the dietary supplementation with DGP did not induce significant variations in the carcass yield. Regarding the pH values of meat samples (45 min and 48 h post mortem) no variations were evidenced (*p* > 0.05), as well as no significant differences were highlighted for drip loss and cooking loss. Additionally, in the case of the meat color (Table 3), the variables L*, a*, and b* did not undergo variations following the administration of the experimental diet (*p* > 0.05). 

Similarly, the dietary supplementation with DGP did not even affect the chemical composition of meat samples, since no significant differences were observed in terms of moisture, total lipids, and ash (*p* > 0.05).

### 3.2. Fatty Acid Profile and Lipid Oxidation Status

The evaluation of the fatty acid profile in DGP (Table 1) showed a remarkable content of linoleic acid (C18:2 *cis*-9 *cis*-12), which accounts for about the 70% of the total fatty acids identified in the by-product. This data certainly had a direct effect in inducing a significant increase in linoleic acid in the diet administered to the experimental group (*p* < 0.05). No significant variations were evidenced for the other fatty acids identified in calf diets, namely palmitic acid (C16:0), stearic acid (C18:0), oleic acid (C18:1 *cis*-9), and linolenic acid (C18:3 *cis*-9 *cis*-12 *cis*-15).

The evaluations performed on meat (Table 4) showed a significant increase in the linoleic acid concentration as a consequence of the DGP dietary intake (*p* < 0.05). In addition to linoleic acid, the only identified PUFA was linolenic acid, which, however, accounts in all samples for less than 0.50 %; for this reason, the marked increase in C18:2 also led to a significant increase in total PUFA (*p* < 0.05). No significant changes were instead reported in the case of SFA and MUFA (*p* > 0.05).

Another finding of the experimentation was certainly due to the determination of the oxidative status of meat samples stored at 4 °C for 3 and 7 days post mortem. After 3 days, the levels of malondialdehyde (MDA) were significantly higher in CG meat samples compared to the EG (0.89 ± 0.03 µg MDA/g vs. 0.65 ± 0.03 µg MDA/g in CG and EG respectively; *p* < 0.05). After a total storage of 7 days, the oxidation increased, as expected, in all the samples, but enhanced the difference between CG and EG meat samples; specifically, the MDA levels in the EG samples were found to be about the half of those found in the control group (2.14 ± 0.14 μg MDA/g vs. 1.01 ± 0.05 in CG and EG respectively; *p* < 0.01). 

### 3.3. Evaluation of Volatile Compounds in Meat Samples

Fifteen volatile compounds (VOCs) were detected in 7 days post mortem meat samples with few differences between the two experimental groups (Table 5), while the adopted methodology did not highlight any compound in the samples taken after 3 days of preservation at 4 °C of the carcass. 

The compound characterized by the highest concentration was an aldehyde, the hexanal, which stood at significantly lower values in meat samples obtained from animals fed the dietary DGP supplementation (*p* < 0.05). The only other difference was found in the family of ketones, in which the 2,3-octanedione significantly increased in the EG samples (*p* < 0.05).

### 3.4. Determination of Biogenic Amines

The HPLC analysis of biogenic amines was based on the purpose of highlighting the presence in meat of tryptamine, 2-phenethylamine, putrescine, cadaverine, serotonin, and tyramine. In the samples collected after 3 days post mortem it was not possible to identify any of the studied amines. Only in the meat obtained in the subsequent sampling were two amines identified in both CG and EG, specifically putrescine and tyramine, although their concentration was lower than the limit of quantification (LOQ).

## 4. Discussion

The present research aimed to evaluate the effect of a diet containing 10% DGP on the qualitative aspects of beef, with particular attention to the fatty acid composition, the oxidative stability, the aromatic profile, and the potential accumulation of biogenic amines. 

The DGP supplementation did not significantly affect feed intake, life weight, carcass weight, and the carcass yield, presumably due to the absence of variations in the energy balance of diets. These results are in agreement with what has been previously reported by Moote et al. (2014) who tested on steers a dietary supplementation with fermented winery by-product [21]; similar results were obtained also on lambs, although in this case authors highlighted an increase of bone weight after a dietary supplementation of 10% GP, a finding that was justified, at least in part, by the flavonoids ability to enhance osteoblasts differentiation and inhibit the osteoclasts function [22].

Values of pH in muscle (pH_45_ and pH_48_) were in the normal range for all the animals involved in the study as well as drip loss and cooking loss, with no significant difference between the two experimental groups. The dietary intake of antioxidants, such as vitamin E, was reported to induce several benefits on drip loss, probably due to the ability of these compounds to stabilize the mitochondrial membranes through the inhibition of phospholipase A_2_ activity [23]. It is well known that GP is rich in compounds that have the capacity to act as powerful antioxidants by scavenging free radicals and terminating oxidative reactions [24]; probably in this experimentation none of these compounds had direct effects on the biochemical mechanisms that influence the tissue’s ability to retain water. 

Color is a highly variable parameter which affects consumer decisions concerning the meat purchase [25]. When comparing beef meat color, no statistically significant differences were observed between the two groups. A similar finding was evidenced in the study of Moote et al. (2014) on the effect of fermented winery by-products supplemented in the bovine diet [21]. In a study on chicken breast meat no significant differences in muscle color lightness and yellowness were observed after GP inclusion in the diet, however a relevant variation was highlighted for redness, with paler meat as a consequence closely related to the experimental feeding strategy; the authors postulated that variations in meat color may be influenced by the presence in grapes of anthocyanin pigments, whose content is strongly influenced by variety and dosage of the grape [26,27]. 

Additionally, with regard to the chemical composition of meat samples, the dietary DGP supplementation did not induce significant variations, confirming the evidence previously reported in a similar study on steers, conducted by Moote et al. (2014) [21]. 

In the last years, growing interest has been developed on the identification of feeding strategies aimed at manipulating the fatty acid composition of cattle meat. This is because animal fats, excluding fish lipids, generally contain a high proportion of SFA, which have been associated to several diseases [28]. Fat tissue in ruminants differs from the single-stomached species because of the higher proportion of SFA and lower proportion of PUFA. This is the direct consequence of the ruminal biohydrogenation mechanism which leads to the conversion of dietary PUFA into SFA or unsaturated fatty acids with fewer double bonds [29]. In this study, a significant increase in concentration of PUFA was observed in the EG samples. This finding is certainly justified by the marked increase of linoleic acid in the same samples, most likely due to the greater concentration of this compound in grape pomace, therefore in the experimental diet. Furthermore, the grape pomace could have introduced in the EG diet bioactive compounds capable of slowing down or blocking the biohydrogenation process mediated by ruminal microorganisms [5]. This result has certainly led to an increase in the PUFA:SFA ratio, which is generally low in ruminants compared to the most favorable balance found in pork and poultry meat [29].

The fatty acid composition found in meat has direct effects on its oxidative stability; generally, higher concentrations of PUFA make meat more susceptible to oxidation, with potentially negative effects on flavor, color, texture, and nutritive value [28]. In this study, both after 3 and 7 days of storage, the EG meat samples showed a reduced lipid oxidation compared to the CG samples, witnessing a direct effect of dietary DGP supplementation in limiting this phenomenon, despite the higher PUFA content. The finding concerning the reduction of lipid peroxidation through dietary GP supplementation has been already observed in chicken meat. In particular a role of dietary GP intake in inducing the increase of liver vitamin E content was reported as a consequence of the sparing effect of GP on vitamin E in the intestine. It has been reported that the 1-electron reduction potential of several polyphenols, such as quercetin, (−)-epicatechin, and (+)-catechin, may reduce vitamin E degradation, resulting in greater amounts of the absorbed compound. Therefore, the feedstuffs supplementation with matrices rich in bioactive compounds may improve vitamin E status in tissues with consequent reduction in lipid oxidation [30]. 

Volatile compounds in meat have been widely studied for their effects on meat flavor [31]. Many factors are involved in the accumulation of VOC in animal tissues, and among them animal diet plays a pivotal role. Most of the study on meat products are focused on cooked meat, which implies that most of the identified VOC originate via heat induced reactions, such as the Maillard or the Strecker reactions [32,33]. In the present study, we chose to analyze VOC from the *longissimus dorsi et lumborum* muscle in order to obtain information more related to the feeding strategy. 

In raw meat sampled after 7 days post mortem 15 compounds were detected both in CG samples and in samples obtained from animals fed the dietary DGP supplementation. The majority of the identified VOC were classified as aldehydes, ketones, and alcohols, and among them hexanal and 2,3-octanedione differed significantly between the two groups. As previously reported by Ford et al. (1976), a feeding strategy containing high levels of linoleic acid seems to be decisive for the accumulation of higher percentages of aldehydes in beef, as a result of an autoxidation mechanism to which the fatty acid is particularly susceptible [34]. In this study, a higher amount of hexanal was observed in the CG samples, therefore it appears as a contradiction since the EG meat samples were richer in linoleic acid, but the concept described above can be reiterated on the role of GP bioactive compounds in slowing down the oxidative processes affecting the lipid component. In a study conducted on cooked pork, Shahidi and Pegg (1994) observed that during the first 6 days of storage, contribution of hexanal to the total volatile aldehydes increased linearly by 650%, after which, its concentration declined quite markedly [35]. Authors also indicated hexanal to be a reliable index of flavor deterioration during the early stages of meat storage, therefore the finding observed in this study sounds particularly interesting, although no evaluations have been made on cooked meat. The other significant difference concerned the increase in the EG samples of 2,3-octanedione, which was generated by an enzymatic reaction mediated by the lipoxygenase that exploits linoleic and linolenic acids as substrates. Since the lipoxygenase is absent in corn and more represented in leafy plants, the abundance of 2,3-octanedione in meat and milk of small ruminants represents an excellent marker of the feeding system [36]. Kanner (1994) reported significant accumulations of 2,3-octanedione, pentanal, and hexanal in roast beef during storage at 4 °C as an effect of lipid peroxidation, therefore through a process which was probably alternative to the enzymatic mechanism just described [37]. In the present study a general improvement of the lipid oxidative stability in the EG samples was evidenced, also evidenced by the reduction of hexanal, therefore it is plausible that animals fed the dietary DGP supplementation have obtained a higher concentration of lipoxygenase as well as greater concentration of its substrate, the linoleic acid, determining already at a ruminal level an increase of 2-3 octanedione. 

Biogenic amines tend to accumulate in foods as a result of the action of several microorganisms, therefore the identification of such compounds might serve as a useful indicator of food spoilage and food safety [38]. High levels of biogenic amines are considered unhealthy, as they can cause pharmacological, physiological, and toxic effects in organisms [39]. Tyramine, cadaverine, putrescine, and histamine are the most prevalent biogenic amines in meat and its derived products [40]. The only amines identified in this study after 7 days post mortem, without significant differences between the two groups, were putrescine and tyramine, whose accumulation can be influenced by the handling and storage of meat [40]. The few and the low amount of biogenic amines identified in our study may be due to both proper handling of the beef carcasses and the effect of bioactive compounds of GP that are known to limit microbial growth and the formation of biogenic amines [41]. However, in this study we did not investigate the effect of DGP phenolic compounds on the biochemical mechanisms that govern bacterial growth, so this aspect deserves further and more specific evaluations.

## 5. Conclusions

In this study was demonstrated that grape pomace inclusion in calves diet resulted in a significant increase of linoleic acid concentration in meat, a condition predisposing to a positive increase in the PUFA:SFA ratio, which is generally low in ruminants compared to the most favorable balance found in pork and poultry meat. Furthermore, an interesting improvement in the oxidative stability of meat samples was evidenced, presumably as an effect of the antioxidant activity performed by the bioactive compounds of which the grape pomace is rich in. This finding was also confirmed by the significant reduction in the same samples of hexanal, a compound whose presence is generally associated with oxidative mechanisms. Overall, the present study showed a viable way for the recovery and the valorization of the main by-product of the oenological industry, although further evaluation should be performed in order to clarify the biochemical mechanisms underlying the observed phenomena.

## Figures and Tables

**Table 1:** Chemical composition of alfalfa hay administered to all animals involved in the study.

Parameters	%
Dry matter (DM)	87.5
Crude protein ^†^	16.3
Ash ^†^	9.1
Ether extract ^†^	2.6
Neutral detergent fiber (NDF) ^†^	53.1
Acid detergent fiber (ADF) ^†^	40.5

^†^ Data are reported on a dry matter basis.

**Table 2:** Ingredients and chemical composition of the custom-formulated concentrate respectively administered to control group (CG) and experimental group (EG).

Ingredients (%)		Diet	
		CG	EG
Beet pulp, dried		11	1
Corn		8.5	8.8
Grain dust		15	9
Grape pomace meal			10
Fine bran		20	22
Sunflower seed		1.5	1.5
Barley		30	34
Soybean meal		4.2	4.9
Molasses		1	1
Soybean hull		3.5	2.5
Mineral premix		2.5	2.5
Commot salt		0.8	0.8
Sodium bicarbonate		1	1
Vitamin Premix		1	1
**Chemical composition (%)**	**DGP**	**CG**	**EG**
Dry Matter (DM)	90.43	90.20	90.44
Crude protein ^†^	12.45	14.80	14.03
Ether extract ^†^	4.02	3.81	4.00
Neutral detergent fiber (NDF) ^†^	38.76	20.33	18.97
Acid detergent fiber (ADF) ^†^	37.53	7.89	8.27
Starch ^†^	nd	29.01	28.69
Ash ^†^	11.08	7.46	7.77
TPC ^‡^ (GAE mg/g)	15.87 ± 1.33	nd	0.24 ± 0.03
**Fatty acids ^§^**	**DGP**	**CG**	**EG**
C16:0	9.97 ± 0.86	21.54 ± 1.44	19.28 ± 1.55
C18:0	2.16 ± 0.22	2.36 ± 0.19	1.98 ± 0.19
C18:1 *cis*-9	15.56 ± 1.34	19.27 ± 1.60	17.55 ± 1.41
C18:2 *cis*-9 *cis*-12	71.59 ± 5.03	53.11 ± 2.01 ^a^	58.31 ± 2.85 ^b^
C18:3 *cis*-9 *cis*-12 *cis*-15	0.22 ± 0.03	1.37 ± 0.12	1.09 ± 0.11

^†^ Data are reported on a dry matter (DM) basis. ^‡^ Total phenolic compounds (TPC) are reported as Gallic Acid Equivalent (mg/g). ^§^ Data are expressed as mean percentage on total fatty acid methyl esters (FAME) ± standard deviation. ^a,b^ Means with different superscripts are significantly different by diet (*p* < 0.05). DGP = dried grape pomace; nd = not detectable.

**Table 3:** Physical and chemical evaluations on meat samples obtained from calves fed a standard diet (CG) and calves fed a dietary supplementation of 10% dried grape pomace (EG).

	Diet		
Trait	CG	EG	*p*
Live weight, kg	328 ± 44	350 ± 22	ns
Carcass weight, kg	183 ± 26	196 ± 15	ns
Carcass yield, %	55.83 ± 0.82	55.91 ± 1.23	ns
pH_45_	6.36 ± 0.03	6.38 ± 0.08	ns
pH_48_	5.55 ± 0.02	5.55 ± 0.06	ns
Drip loss, %	2.64 ± 1.23	3.25 ± 1.57	ns
Cooking loss, %	22.36 ± 2.90	22.40 ± 8.83	ns
L*	41.89 ± 4.22	40.20 ± 4.37	ns
a*	18.15 ± 2.63	17.94 ± 2.55	ns
b*	12.55 ± 2.51	12.64 ± 1.79	ns
**Chemical composition (%)**			ns
Moisture	74.53 ± 0.98	75.11 ± 1.90	ns
Dry matter ^†^ (DM)	25.47 ± 0.98	24.89 ± 1.90	ns
Total lipids ^†^	1.23 ± 0.13	1.10 ± 0.09	ns
Ash ^†^	1.09 ± 0.12	0.95 ± 0.91	ns

All data are reported as mean ± standard deviation; ns: not significant. pH_45_: pH 45 min post mortem; pH_48_: pH 48 h post mortem. L*: lightness; a*: redness; b*: yellowness. ^†^ Data are reported on a dry matter basis.

**Table 4:** Fatty acid profile of meat samples obtained from animals fed a standard diet (CG) and animals fed a dietary supplementation of 10% dried grape pomace (EG).

Fatty Acids ^†^	CG	EG	*p*
C14:0	2.37 ± 0.68	2.68 ± 0.62	ns
C15:0	0.39 ± 0.06	0.42 ± 0.06	ns
C16:0	26.56 ± 2.59	27.53 ± 1.55	ns
C17:0	1.24 ± 0.20	1.02 ± 0.31	ns
C18:0	18.64 ± 0.27	16.33 ± 0.20	ns
C22:0	1.79 ± 0.57	1.75 ± 0.88	ns
SFA	50.99 ± 3.07	49.74 ± 2.06	ns
C14:1	0.31 ± 0.15	0.33 ± 0.13	ns
C16:1	1.98 ± 0.62	1.82 ± 0.49	ns
C18:1 *cis*-9	25.79 ± 1.35	26.20 ± 1.48	ns
C18:1 *trans*-11	1.74 ± 0.35	1.55 ± 0.29	ns
C18:1 *trans* ^‡^	6.64 ± 3.28	4.13 ± 2.74	ns
MUFA	36.45 ± 2.78	33.96 ± 3.25	ns
C18:2 *cis*-9 *cis*-12	8.76 ± 1.47	13.03 ± 1.98	*
C18:3 *cis*-9 *cis*-12 *cis*-15	0.39 ± 0.11	0.43 ± 0.24	ns
PUFA	10.94 ± 1.42	15.21 ± 3.01	ns

^†^ Data are reported as mean percentage on total FAME ± S.D. ^‡^ Sum of *trans* isomers with the exception of the *trans*-11 reported separately. SFA: saturated fatty acid; MUFA: monounsaturated fatty acid; PUFA: polyunsaturated fatty acid. * *p* < 0.05; ns: not significant.

**Table 5:** Volatile compounds (VOCs) detected after 7 days post mortem in beef samples obtained from animals fed a standard diet (CG) and animals fed a dietary supplementation of 10% dried grape pomace (EG).

VOC ^†^	CG	EG	*p*
pentanal	1.91 ± 0.24	1.99 ± 0.23	ns
hexanal	56.24 ± 3.31	45.95 ± 4.05	*
heptanal	2.84 ± 0.38	2.97 ± 0.34	ns
octanal	2.82 ± 0.42	2.54 ± 0.36	ns
nonanal	6.55 ± 0.57	7.61 ± 0.82	ns
2-octenal	0.33 ± 0.05	0.28 ± 0.04	ns
2-heptanone	0.30 ± 0.04	0.44 ± 0.05	ns
2,3-octanedione	8.98 ± 0.91	13.20 ± 1.67	*
1-pentanol	0.96 ± 0.05	1.36 ± 0.16	ns
1-octen-3-ol	11.43 ± 1.04	15.03 ± 1.79	ns
2-octen-1-ol	2.29 ± 0.24	2.59 ± 0.17	ns
ethylbenzene	0.38 ± 0.05	0.31 ± 0.04	ns
β-Terpinyl acetate	2.48 ± 0.34	3.23 ± 0.35	ns
benzaldehyde	1.84 ± 0.19	1.92 ± 0.19	ns
furan, 2-pentyl	0.65 ± 0.08	0.58 ± 0.06	ns

^†^ Data are reported as mean percentage of (VOCs) ± S.D. * *p* < 0.05; ns: not significant.
